# Alternative ways of representing Zapotec and Cuicatec folk classification of birds: a multidimensional model and its implications for culturally-informed conservation in Oaxaca, México

**DOI:** 10.1186/1746-4269-9-81

**Published:** 2013-12-09

**Authors:** Graciela Alcántara-Salinas, Roy F Ellen, Leopoldo Valiñas-Coalla, Javier Caballero, Arturo Argueta-Villamar

**Affiliations:** 1 Centro Regional de Investigaciones Multidisciplinarias, Universidad Nacional Autónoma de México. UNAM, Av. Universidad s/n, circuito, Colonia Chamilpa, Campus Morelos, Cuernavaca, Morelos C.P. 62210, Mexico; 2 Centre for Biocultural Diversity, School of Anthropology and Conservation, University of Kent, Marlowe Building, Canterbury, UK; 3 Instituto de Investigaciones Antropológicas, Universidad Nacional Autónoma de México. UNAM. Circuito Exterior, Ciudad Universitaria, Coyoacán C.P. 04510, D.F, Mexico; 4 Jardín Botánico Exterior, Instituto de Biología, Universidad Nacional Autónoma de México. UNAM, Circuito Exterior s/n, Ciudad Universitaria, Copilco, A.P. 70-614, Coyoacán Distrito Federal C.P. 04510, Mexico; 5 Centro Regional de Investigaciones Multidisciplinarias, Universidad Nacional Autónoma de México. UNAM, Av. Universidad s/n, circuito, Colonia Chamilpa, Campus Morelos, Cuernavaca, Morelos C.P. 62210, Mexico

**Keywords:** Animal conservation, Ethno-ornithological knowledge, Folk classification, Oaxaca, Mexico

## Abstract

**Background:**

We report on a comparative ethno-ornithological study of Zapotec and Cuicatec communities in Northern Oaxaca, Mexico that provided a challenge to some existing descriptions of folk classification. Our default model was the taxonomic system of ranks developed by Brent Berlin.

**Methods:**

Fieldwork was conducted in the Zapotec village of San Miguel Tiltepec and in the Cuicatec village of San Juan Teponaxtla, using a combination of ethnographic interviews and pile-sorting tests. Post-fieldwork, Principal Component Analysis using NTSYSpc V. 2.11f was applied to obtain pattern variation for the answers from different participants.

**Results and conclusion:**

Using language and pile-sorting data analysed through Principal Component Analysis, we show how both Zapotec and Cuicatec subjects place a particular emphasis on an intermediate level of classification. These categories group birds with non-birds using ecological and behavioral criteria, and violate a strict distinction between symbolic and mundane (or ‘natural’), and between ‘general-purpose’ and ‘single-purpose’ schemes. We suggest that shared classificatory knowledge embodying everyday schemes for apprehending the world of birds might be better reflected in a multidimensional model that would also provide a more realistic basis for developing culturally-informed conservation strategies.

## Abstract (es)

**Abstracto:**

**Antecedentes**

Se presenta un estudio etno-ornitológico comparativo de dos comunidades indígenas del Norte de Oaxaca, México, una Zapoteca y otra Cuicateca, que desafía algunas de las descripciones existentes hasta ahora de las taxonomías folk. El modelo usado por default fue el sistema taxonómico de rangos desarrollado por Brent Berlin.

**Métodos**

La investigación de campo se realizó en San Miguel Tiltepec comunidad Zapoteca y San Juan Teponaxtla comunidad Cuicateca, los datos etnográficos se obtuvieron a través de entrevistas y sorteo de cartas. Posterior al trabajo de campo se realizó un análisis de Componentes Principales usando el programa NTSYSpc. V.2.11f para obtener patrones de variación en las respuestas obtenidas de las diferentes participantes.

**Resultados y conclusión**

Mediante la evidencia del uso del lenguaje y el análisis de técnicas fenéticas como el Análisis de Componentes Principales aplicadas a datos obtenidos por ‘sorteo de cartas’, se muestra como los sujetos entrevistados en ambas comunidades tienden a colocar los ‘ítems’ utilizados en un nivel intermedio de clasificación. Las categorías obtenidas agrupan del mismo modo aves con no-aves utilizando criterios ecológicos y de comportamiento, lo cual infringe con una estricta distinción entre lo simbólico y ordinario (o natural), y entre aquellos esquemas de propósitos generales y propósitos únicos. Este sistema clasificatorio expresa esquemas habituales para comprender el mundo de las aves y el cual puede ser reflejado de mejor manera mediante un modelo multidimensional de clasificación, el cual, a su vez ofrece bases bien sustentadas para generar estrategias de conservación que integren aspectos culturales.

## Background

Over the last 50 years, the study of plant and animal folk classification has provided important evidence for understanding the logic and meaning of the processes by which cultural categories are established more generally. By categories we mean here those entities that the human mind creates in order to make sense of the diversity of experience, by grouping things, attributes and phenomena on the basis of similarity and difference; and by classification, the ways in which categories are related to each other, and the means by which particular cultural patterns are produced [1]. Since the early path-breaking work of Conklin [2] several directions have developed in the study of the folk classification of living things, which have focussed on a number of different theoretical issues. Five of these are highlighted by Zent [3]: a) universality versus relativity, b) intellectualist versus utilitarian motives, c) taxonomy versus fuzzy sets, d) general purpose versus special purpose classifications, and e) cultural models versus individual contextual schemata. Let us examine each of these in turn.

(a) The debate juxtaposing universality and relativity is associated with the view that the underlying principles, and to some extent the actual categories, evident in different ethnobiological classification systems, reflect universal properties of the human mind. The main proponents here include Cecil Brown [4], Brent Berlin, James Boster and Scott Atran. Berlin [5] provides us with evidence and makes claims for widespread regularities concerning plant and animal categorisation and systems of ethnobiological knowledge organisation across cultures, concluding that underlying similarities reflect a universal human pattern and a common developmental sequence. Both Boster [6] and Atran [7] have progressed Berlin’s approach theoretically and methodologically, connecting it more obviously with current work on human cognition conducted by psychologists. This work in turn has made possible claims that pan-human regularities in the organisation of natural history knowledge might support the idea that the human mind is ‘modular’. However, models of mental modularity in the human brain build on generalizations concerning a pre-linguistic phase of cognitive development and assume a degree of genetic determination or ‘hard-wiring’ [8]. The relativists, by contrast, argue that many aspects of these systems most likely reflect local ecological variation and varying cultural representations and uses [9-16], and insist that many (though not necessarily all) ‘universals’ simply reflect the converging common experience of different groups. As Descola [17] has argued, it may now be necessary to go beyond simplistic notions of universality and relativism in trying to make sense of ethnobiological classification.

(b) Utilitarian versus intellectualist motivations. During the formative phase of the development of ethnobiological studies, from the late nineteenth to the mid-twentieth century, the main focus was on the utilization of plant and animal life [13,18], without any real interest in the cognitive aspects. In the work of Lévi-Strauss (through structuralism) and that of Conklin (through ethnoscience) an interest developed in the organisation of ethnobiological knowledge independent of the material uses to which it was put. However, it was this development which itself generated opposing claims as how to explain the observed cognitive patterns inferred from folk classification data. On the one hand, there were those who argued for a utilitarian approach to folk classification, viewing names and classifications of living things as a reflection of mainly practical concerns, while on the other hand there were those who argued for an intellectualist approach, and who tended to emphasise the way names and classifications emerge through an autonomous mental process inherent in shared human cognition, and subject to pressures of natural selection [16,19].

(c) Taxonomy versus fuzzy sets. This debate is associated with the view, first formulated by Berlin and his colleagues [5,20], of the pan-cultural universality of the idea of taxonomic hierarchy, meaning classification operating through the logic of class inclusion, contrast and ranking. Others, including Hunn [1,11,21], have suggested that ethnobiological classifications are in practice often characterised by flexibility and fuzzy logic, and that the taxonomic model may be a misleading guide to how classificatory knowledge is generally stored, retrieved and utilised in oral folk cultures.

(d) General-purpose versus special-purpose classifications. This distinction was introduced by Berlin as part of his argument favouring a universal taxonomic model underlying ethnobiological classifications based on ‘natural’ discontinuities, which ‘carve nature at the joints’ [22]. Those who have argued against this [1,23], claim that, in practice, people combine aspects of special purpose and general purpose constructs depending on circumstances, and that to distinguish one from the other is too rigid. These same critics argue that a generalization of rank based on abstract properties is inconsistent with a holistic and dynamic conception. The position of one category in relation to others much depends on context. Berlin’s idea of a pre-eminent ‘natural’ general-purpose classification also requires excluding symbolic or ritual classifications and placing them in a particular kind of special-purpose classification. However, the evidence of sorting tests and other methodologies suggest that in many contexts people do not distinguish systematically between general-purpose and special-purpose or between the social and non-social worlds, and - in practical terms - boundaries between these are often unclear. Metaphorical and symbolic thought are central to human cognition of the material world. Symbolic things are in an important sense practical, and practical classifications of the non-social world often rely on metaphors that are ultimately social, as in the use of the terms ‘genus’ and ‘family’ to organise plants and animals [1].

(e) Shared cultural models versus individual contextual schemata. The work of Berlin shows awareness of the problem of ‘the omniscient-speaker hearer’, that is the tendency of ethnographers – sometimes unwarrantedly - to assume a sufficiently high level of cultural sharing to justify statements of the kind ‘the Zapotec believe that’, ‘the Cuicatec know’, etc. This is often sustained by relying on exceptional individual informants with extensive knowledge [24]. Although all populations require a level of shared cultural knowledge to be effective socially, much of this is distributed and varies according to context [25]. There are now many attempts to measure intra-cultural variation and disagreement among informants through cultural consensus analysis and other methodologies [25-27].

Depending on the context, a folk classification is instantiated in different ways, conforming to the notion of ‘prehension’ put forward by Ellen, Thus:

people bring to situations in which classifying activity takes place, and from which verbal statements about classifying behaviour result, information of diverse kinds acquired through both informal and formal socialisation experience, of the world in general and of earlier classifying situations. How they then classify depends upon the interplay of this past knowledge (including prescriptions and preferences with regard to particular cognitive and linguistic idioms) with the material constraints of the classifying situation, the purposes of the classifying act, and upon the inputs of others [28].

The work conducted by Alcántara-Salinas on the ethno-ornithological knowledge of the Zapotec and Cuicatec in northern Oaxaca has raised some of these classical theoretical issues in an acute way, and in particular in terms of which modes of representing the natural world are most relevant when seeking to make connections between local knowledge systems and effective conservation practice. The multidimensional model developed in connection with her comparative study [29] shows how we might accommodate both symbolic and mundane, general-purpose and single-purpose criteria, and the different kinds of context which modify classifying behaviour. By applying this methodology to data from two field sites, one Zapotec, one Cuicatec, we further evaluate the validity of the model in relation to the notion of ethno-taxonomic hierarchy formulated by Berlin and his colleagues.

## Methods

**Figure 1:**
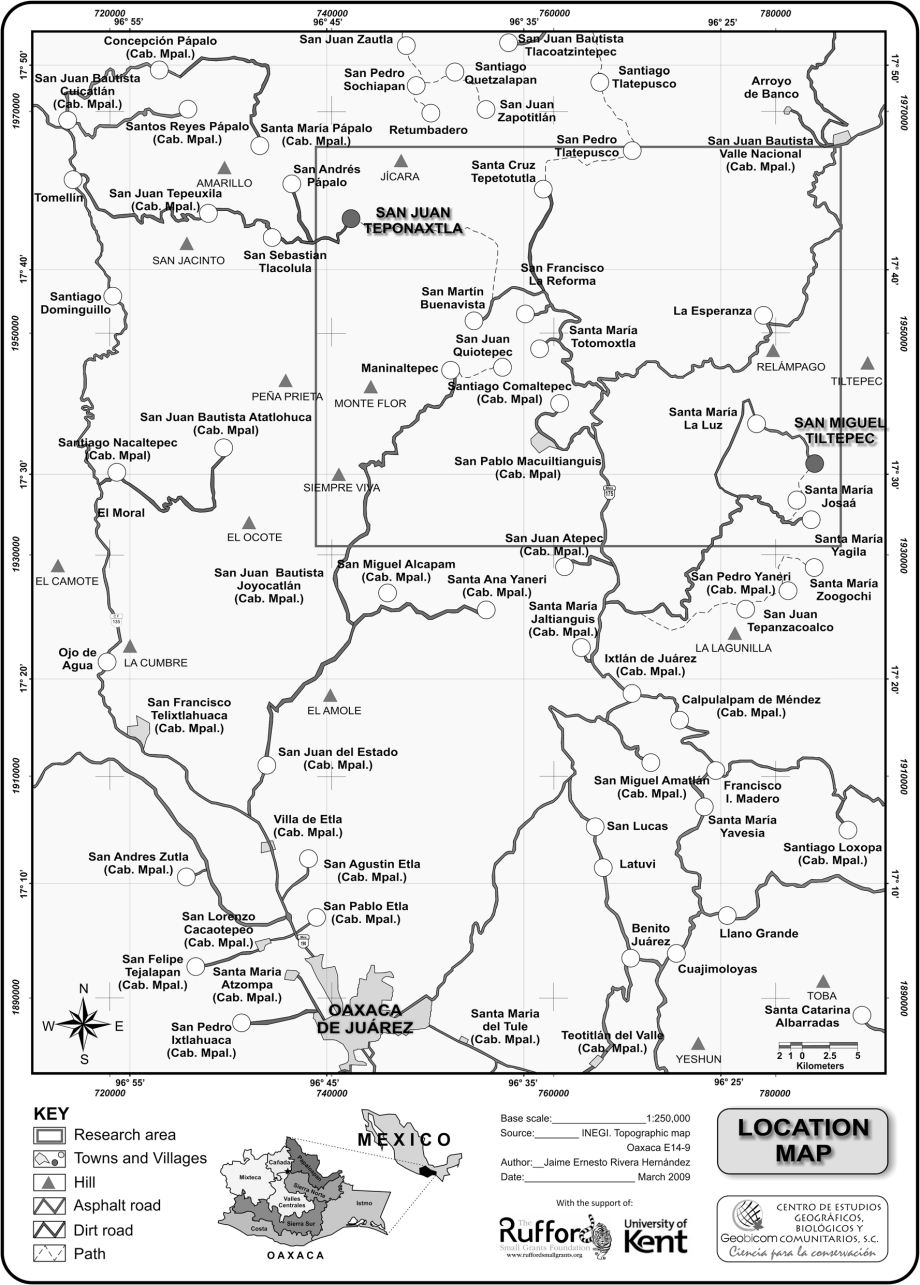
**Location of San Miguel Tiltepec and San Juan Teponaxtla in Northern Oaxaca.** The two villages are approximately 45 km apart.

The research reported here is based on two periods of fieldwork, the first conducted in the Zapotec cultural area in the Sierra Norte region (San Miguel Tiltepec) from 1997–2003, and the second in the Cuicatec cultural area bordering the Cañada region (San Juan Teponaxtla) from 2007 to 2008 (Figure 1). Zapotec and Cuicatec are both Otomanguean languages [30,31]. The Proto-Otomanguean ‘homeland’ was in the Tehuacán Valley, in Puebla, and probably other places where we find the same cultural sequence, representing the Coxcatlán Phase (5000–3400 B. CE). Diversification appears to have taken place in parallel to the development of agriculture [32]. Both San Miguel Tiltepec and San Juan Teponaxtla are characterised by high a degree of biocultural richness across a series of habitats, including rain forest, cloud forest, pine-oak forest, pine forest, semi-deciduous forest, and thorn forest. Although there is a lack of inventory studies, current estimates suggest combined species figures for both regions of almost 117 mammals [33], 736 birds [34], 133 amphibians [35], 247 reptiles [35] and 127 fresh water fish [36]. This rich biodiversity is paralleled by a diversity of agricultural crops in both areas [37-43], with a rich linguistic diversity well attested for Oaxaca as a whole [44].

The Mexican National Institute of Indigenous Languages has reported 410,906 speakers of Zapotec and 12,610 speakers of Cuicatec in Oaxaca as a whole in 2009 [45]. In 2002 there were 290 speakers of Zapotec in San Miguel Tiltepec [46] and 87 speakers of Cuicatec in San Juan Teponaxtla [46]. During fieldwork Alcántara-Salinas used structured interview techniques within a wider portfolio of methods. In the circumstances (asymmetric indigenous language competence and high rates of language erosion) researcher and participants communicated in Spanish, which had the additional advantage of easing the standardization of question protocols, of being the language of official discourse, and of conservation policy and practice in particular. However, full data were assembled on Zapotec and Cuicatec folk names for categories applied to birds, at all degrees of inclusiveness, and with identifications to the phylogenetic species level. Categories were inferred from language use, using the default Berlin model. The intermediate categories identified and examples of their content in terms of both folk names and scientific species are presented in Tables 1, 2 and 3, and these are discussed in the results section below.

**Table 1:** A quantitative comparison of some features of Zapotec and Cuicatec folk classification of birds

	**Zapotec**	**Cuicatec**
Phylogenetic species	209	227
Intermediate folk groups	4	6
Folk generics	30	36
Folk specifics	77	69
Folk varietals	11	9
Overlap between the content of different intermediate groups for all folk terms	6	4
Synonyms	78	93

**Table 2:** Indicative data illustrating the allocation of vernacular names and their scientific equivalents by Zapotec research subjects

**Intermediate labelled categories**	**Total Zapotec names reported**	**Total scientific species equivalents**	**Selected examples of Zapotec names**	**Scientific taxa**
***bëa artaba rhela***	2	3	*wëlhopa’*	*Ciccaba virgata*
			*chghii*	*Chordeiles acutipennis*
***bëa gishi***	18	12	*chghii*	*Chordeiles acutipennis*
			*bdëu banruko*	*Dactylortyx thoracicus*
			*pato gishi dou*	*Formicarius analis*
			*ptzia’ nia gatho*	*Odontophorus guttatus*
			*bërha bdau*	*Sarcoramphus papa*
			*brhudi gishi*	*Crax rubra*
***bëa lurshba***	20	19	*bërha bdau*	*Sarcoramphus papa*
			*chiraba zopilote*	*Coragyps atratus*
			*p’jia yego*	*Buteogallus solitarius*
			*brhudi gishi*	*Crax rubra*
			*chenchogodiu tupa’*	*Streptoprocne zonaris*
			*vigini win*	*Stelgidopteryx serripennis*
***bëa rhsbaa***	72	175	*bdëu banruko*	*Dactylortyx thoracicus*
			*bdëu xhedeu*	*Claravis pretiosa*
			*ighrhiili xheen*	*Amazona oratrix*
			*ratutzi ladou*	*Lampornis amethystinus*
			*radyeko yaa*	*Aulacorhynchus prasinus*
			*vigini win*	*Stelgidopteryx serripennis*
***Total number of unique names***	112	209		

Folk names and scientific taxa appearing in more than one intermediate category are underlined. The total numbers of folk names and scientific taxa do not include those that are repeated.

**Table 3:** Indicative data illustrating the allocation of folk names and their scientific equivalents by Cuicatec research subjects

**Intermediatelabelled categories**	**Total Cuicatec names reported for category**	**Total scientific species equivalents**	**Selected examples of Cuicatec names**	**Scientific taxa**
***íti nhúnhi***	2	5	*túu* *nhúnhi*	*Cairina moschata*
			*yódo* *nhúnhi*	*Chloroceryle americana*
				*Sayornis nigricans*
				*Sayornis phoebe*
				*Cinclus mexicanus*
***íti ngo nōhō***	10	6	*kúukū’ey*	*Chordeiles acutipennis*
			*kón kurri*	*Antrostomus vociferus*
			*ímhi túu*	*Ciccaba virgata*
			*ímhi íkhiāan*	*Asio stygius*
			*dong’uko*	*Psiloscops flammeolus*
			*ghuanda*	*Psiloscops flammeolus*
			*yódo* *dōondi*	*Chordeiles acutipennis*
***íti yo ‘ínu***	22	15	*túu* *kuáti*	*Dendrortyx macroura*
			*kúukū’ey*	*Chordeiles acutipennis*
			*kón kurri*	*Antrostomus vociferus*
			*‘inhio khuā*	*Crax rubra*
			*túu* *nhúnhi*	*Cairina moschata*
			*‘inhio chiquito*	*Myioborus miniatus*
			*yódo mayata*	*Myioborus pictus*
***nōhōndo***	1	15	*tíind dú*	All Trochilidae
			*yódo* *nōhōndo*	*Phaethornis longirostris*
				*Tilmatura dupontii*
				*Eugenes fulgens*
				*Archilochus colubris,*etc.
***íti ngo yuta***	17	11	*lúti khuá*	*Cathartes aura*
			*íyho pinto*	*Buteo jamaicensis*
			*ínhiúu íkhiāan*	*Micrastur ruficollis*
			*nhiúu*	*Accipiter cooperii*
			*lú’ ka*	*Falco sparverius*
			*salú’ ka*	*Falco columbarius*
***no yun*** ***a***	77	180	*‘iho kiáa*	*Ramphastos sulfuratus*
			*ditō tíi ‘khúhon*	*Campephilus guatemalensis*
			*kukurée*	*Columbina inca*
			*ditāha khúhon*	*Sittasomus griseicapillus*
			*iti ngangui*	*Campephilus guatemalensis*
			*yódo mayata*	*Myioborus pictus*
***Total number of unique names***	124	227		

Folk names and scientific taxa appearing in more than one intermediate category are underlined. The total numbers of folk names and scientific taxa do not include those that are repeated.

In addition, interviews included a pile-sorting test of the kind advocated by Puri [47]. The test involved 33 13 × 13 cm cards, each card representing a selected species or more-inclusive taxon from the range of fauna known to both populations. Each card displayed the photograph of an animal (recorded previously in the area) on the front and a number to identify the phylogenetic status of each species on the back. Twenty-eight persons at each research site were recruited to participate in the pile sorts. The group included both male and female children, adolescents and adults (Table 4), males and females being selected from alternate households. All the tests were conducted, where possible, inside the houses of participants to avoid unnecessary crowding and distractions.

**Table 4:** Age and sex composition of samples used in pile sorting tests conducted with Zapotec and Cuicatec subjects

**Age range**	**Zapotec**			**Cuicatec**		
	**Female**	**Male**	**Total**	**Female**	**Male**	**Total**
Children (6–13)	4	3	7	4	1	5
Young adults (14–40)	5	8	13	2	3	5
Old adults (41–90)	3	5	8	7	11	18
**Total**	**12**	**16**	**28**	**13**	**15**	**28**

Participants were asked to sort the cards into piles based on overall similarity, but were not provided with instructions as to what criteria to use. In a few instances, however, examples were provided where participants requested more information. Participants were then asked to name each pile and to give their reasons for grouping the animals in a particular way. The results for both San Miguel Tiltepec and San Juan Teponaxtla interviews were processed in the same way. We expected participants to make several judgments about animals when making pile-sorts. We were able to differentiate all these judgments qualitatively, after which Alcántara-Salinas codified the qualitative judgments and constructed a 51 (judgments) × 33 (animal card) matrix and a 57 (judgments) × 33 (animal card) matrix respectively. Each cell in the matrix reported the number of times each animal was mentioned in any judgment. For instance, the snail was grouped in a positive relationship to humans by three Zapotec interviewed while only one Cuicatec grouped it in the same judgment. A Principal Component Analysis (PCA) using NTSYSpc V. 2.11f was applied to obtain pattern variation for the answers from different participants [48]. We used the first three components C1, C2, C3, as they explained the higher amount of the variation. As we considered each component as an orthogonal axis in an Euclidian space, it was not possible to display more than three axis at the same time.

The research was approved by the University of Kent School of Anthropology and Conservation Ethics Committee, which works within the Ethical Framework stipulated by the UK Economic and Social Research Council, and within the guidelines of the International Society for Ethnobiology. In Oaxaca, prior informed consent for fieldwork was obtained from the authorities in San Juan Teponaxtla and San Miguel Tiltepec. On completion of the research, oral reports were submitted to the communities concerned. A written report was also submitted in the form of Rivera-Hernández, J.E., G. Alcántara-Salinas and A. Vergara Villamil 2009. Guía ecoturística de la biodiversidad y la cultura de San Juan Teponaxtla, Cuicatlán, Oaxaca, 216 pp. Cordoba, Mexico: Centro de Estudios Geográficos Biológicos y Comunitarios S.C., Rufford Small Grants Foundation and PACMYC-CONACULTA.

## Results and discussion

### Language data

Our language work highlights several features of Zapotec and Cuicatec classificatory schemes that pose problems for the model of ethnozoological classification outlined in our introductory section. When we asked Zapotec and Cuicatec research subjects to group individual bird species within the (folk kingdom) category for animals as a whole, labelled *bëa* (Zapotec) or *íti* (Cuicatec), it became clear that they were using groupings that were not exclusive to birds, and which included insects, bats, flying lizards and flying squirrels among others, and were based on behaviour and habitat attributes as well as morphology. In Tables 1, 2 and 3 we provide some indicative data illustrating the allocation of vernacular names and their scientific equivalents by Zapotec and Cuicatec research subjects respectively. A full list of Zapotec and Cuicatec bird names is provided elsewhere [29].

Alcantara-Salinas recorded 209 bird species for San Miguel Tiltepec, corresponding to 118 Zapotec folk names (Table 2). These were distributed across four named ‘intermediate’ groupings as follows: (1) *artaba rhela* (those living in the night), (2) *gishi* (those that can be seen walking or alighting on the ground), (3) *rhsbaa* (those that fly [low]) and (4) *lurshba* (those flying higher in the sky). In this scheme, categories (2), (3) and (4) are, by contrast with (1), implicitly diurnal. However, these same categories are also applied to other kinds of animal, such as insects, mammals and reptiles. Because these same descriptive terms can be applied to animals other than birds, Zapotec speak of a butterfly as *bëa rhsbaa*, and a worm as *bëa gishi*. Note also that some bird terms and scientific taxa appear in more than one intermediate grouping.

By comparison, we found six intermediate named categories being used in Cuicatec, mapping on to 114 folk names (Table 3). Cuicatec distinguish two groups on the basis of behaviour alone, as follows: (1) *íti nhúnhi*, animals living in the water; and (2) *íti ngo nōhō,* animals living in the night. Diurnal birds are divided into four main groupings, based on behaviour, symbolic features and use. Thus, birds that can be seen walking or alighting on the ground are described as (3) *íti yo ‘ínu, ‘*grass or land animals’, owing to their location in low vegetation habitats; while those birds living near by, or extracting nectar from flowers, are described as (4) *nōhōndo*. Birds seen flying through the canopy of trees or plants are called (5) *no yuna*, ‘flying animals’; while those feeding on meat are called (6) *íti ngo yuta* or ‘meat eating animals’. As amongst the Zapotec, the same descriptive terms can also be applied to kinds of animal other than birds. Thus, a butterfly is *íti no yuna* (a flying animal), while a worm is *íti yo ‘ínu* (a grass or land animal). Similarly, some bird terms and scientific taxa appear in more than one intermediate grouping.

A quantitative comparison of some features of Zapotec and Cuicatec folk classification of birds is provided in Table 1. Note that total numbers differ from Tables 2 and 3 as some species and folk categories are repeated in different intermediate groups. The main word used to name birds is *vigini* in Zapotec and *yódo* in Cuicatec, both terms ranging broadly to include large numbers of both Passeriformes and non-Passeriformes. However, not all birds are *vigini* or *yódo*. For instance, hawks are called *p’jia* or *bugaka* in Zapotec águila or gavilán in Spanish but never *vigini*. Similarly, most Galliformes are *bërha*, parrots are *ighrhiili* in Zapotec, and hummingbirds are *ratutzi* in Zapotec or *tíin dú* in Cuicatec, but never *vigini* or *yódo*. An owl is never a ‘bird’ in this sense either, being labelled *wëlhopa* in Zapotec, or - depending on morphology - either *imhi* or *dong’uko* in Cuicatec. Although the characteristic of flight would seem to be an essential common diagnostic feature when classifying birds in both communities, Zapotec differentiate between ordinary flying birds and birds circling or flying high in the sky, while Cuicatec group both fliers and high fliers together in the same group, *no yuna.* Multiple synonyms are important for both Zapotec and Cuicatec, and this partly relates to the complex overlapping groupings that are found in both cases.

Although intuitively, by comparative inference, and from other data we would expect that birds are also seen by both Zapotec and Cuicatec as a distinctive ‘natural’ group of animals of the kind that would constitute a life form, the prominence of intermediate categories suggests that this level also must be a primary organizing device in folk classification for both Zapotec and Cuicatec. Although the intermediates group animals across the divide between birds and non-birds, utilising broad ecological and behavioural criteria, they cannot easily be described as ‘special purpose’, with the implication that they are some-how secondary or less salient cognitively than general purpose categories. Indeed, they are integral to the way both Zapotec and Cuicatec model the natural world. The extent of synonymy in both languages and complex overlapping groupings suggests that representation of bird classification in terms of two-dimensional taxonomic hierarchies is misleading, and that we need to seek alternatives.

### Pile-sorting data

**Figure 2:**
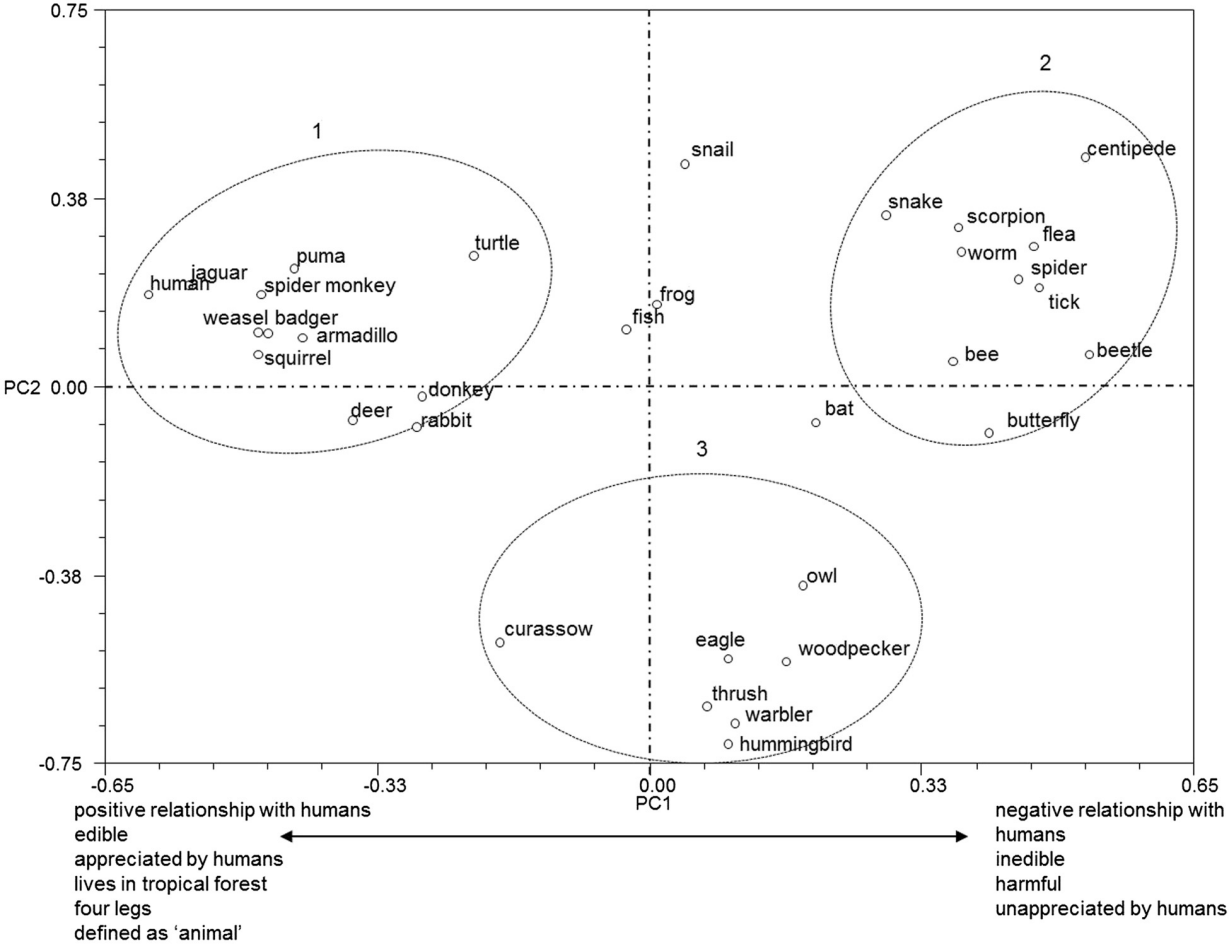
Principal component analysis for all Zapotec judgments in pile-sorting analysis.

The data gathered during the card sorting exercise comprised 268 groupings for Zapotec subjects and 247 groupings for Cuicatec subjects. Zapotec and Cuicatec subjects grouped animals in different ways, on the basis of ‘judgments’ of resemblance relating to different kinds of criteria. In this analysis all judgments have been placed in six main groups. These are: 1) association with humans, 2) behaviour, 3) feeding, 4) habitat, 5) morphological attributes and 6) miscellaneous. As we will show, the judgments are not mutually exclusive and each has a different value. Combining all classificatory judgments made by Zapotec subjects during the pile-sorting exercise we used PCA to generate Figure 2. The first two components accounted for 24.1464 percent of the variation. In examining this figure we can see that on the X axis the first component forms two groups. On the left side of the graph is group 1, based on the Eigen values shown in Additional file 1. These are animals in a positive relationship with humans (S2), being edible or appreciated in other ways, mostly living in tropical forest, having four legs or being defined in neutral terms simply as ‘animals’. On the right hand side of the graph is group 2. These are animals in a negative relationship to humans (S1), being inedible or harmful, or - for example - causing damage by biting. Snails, frogs and fishes are not placed in either group 1 or 2 as they are considered to be aquatic (L1), a classificatory characteristic that is more salient for Zapotec subjects than either positivity or negativity separately. Indeed, the snail is considered to be an animal with neither positive nor negative implications for humans, being an animal of the forest, neutral and with no particular uses.

If we now examine the PC2 for the Y-axis in Figure 2, we can distinguish two groups: groups 1 and 2 on the left side of the graph, and group 3 on the right side of the graph. The separation of group 3 from the other two groups reflects the higher values provided by subjects, as indicated in Additional file 1, namely −0.8243 for flying animals (C3) and −0.8442 for birds (P10). This suggests the criterion of flight as the most important classificatory feature. Among flying animals, the owl is close to the bat because they are both considered omen animals, with a value of −0.6068. The owl is more salient than the others because it is a nocturnal animal, like a bat, and emits sounds, which also places it with the jaguar and puma on both counts, a relationship indicated in Figure 2 by their being placed within the ellipse bounded by a broken line. Additionally, the curassow (*Crax rubra*) is separated from other birds because it does not routinely fly, spending more time walking, and for which reason it is sometimes not considered to be a bird at all.

Additional file 1 shows the importance value of each judgment in each of the first three principal components. The components that contributed more to the first principal component were PC1 and PC2 (4 each), the highest values being those over 0.6, whether negative or positive. For PC1, numbers marked * are the highest: with a value of −0.7241 for character S2 (positive relationship with humans), a value of −0.7247 for character L2 (terrestrial animals which live in the rain forest), a value of −0.7903 for character M10 (animals with four legs), and a value of −0.7903 for character P2, which defines animals as a whole or that ‘just are’ All the highest values - negative or positive - reflect the number of times a particular judgment is mentioned, and the main reasons why people made judgments.

**Figure 3:**
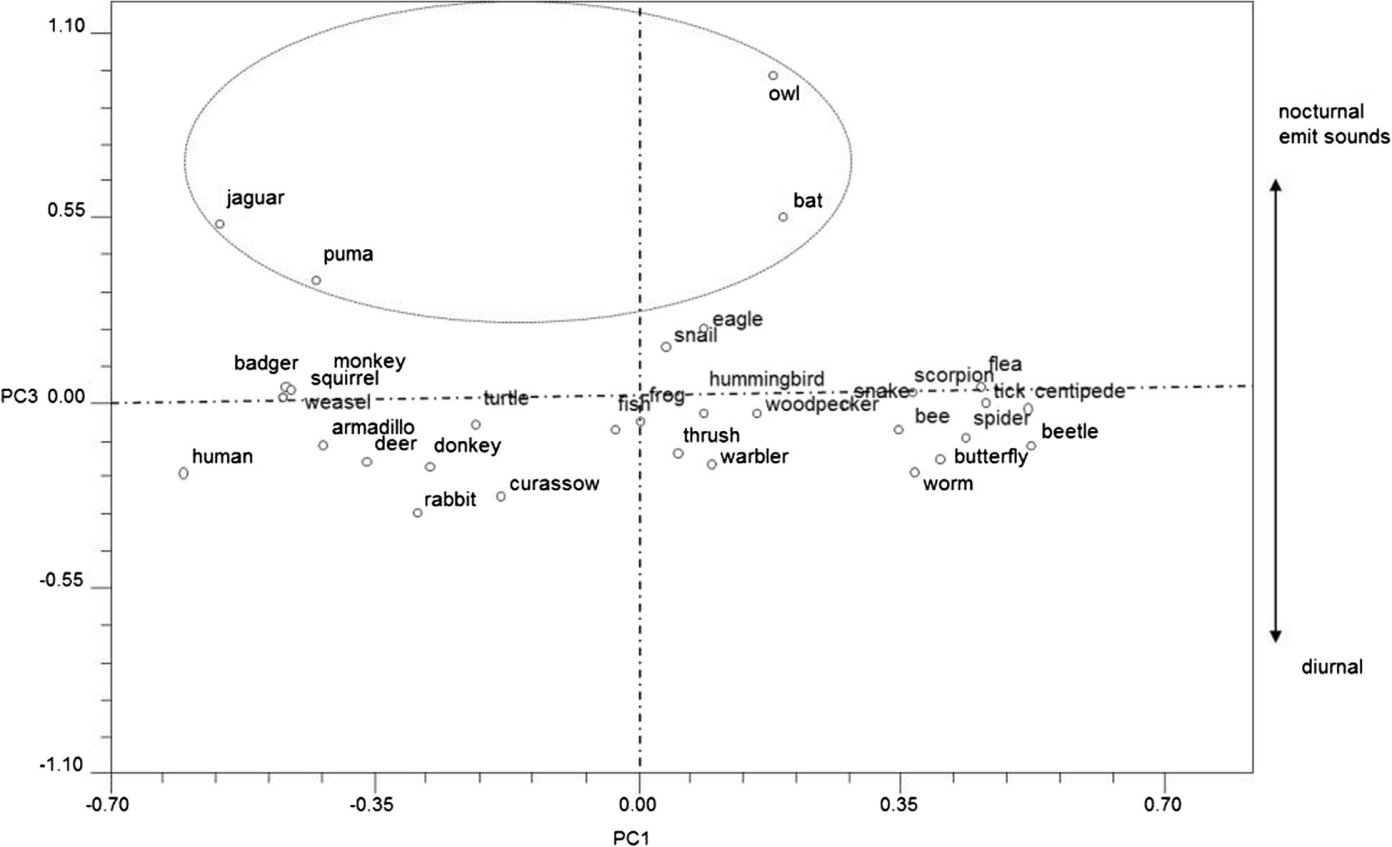
Graph of principal component 3 in Zapotec pile-sorting analysis.

In Figure 3 we can see the graph for PC3. In this case, the values contributing most to explaining variation are 0.8099 for character N (nocturnal animals) and 0.6562 for character Z (animals that emit sounds).

**Figure 4:**
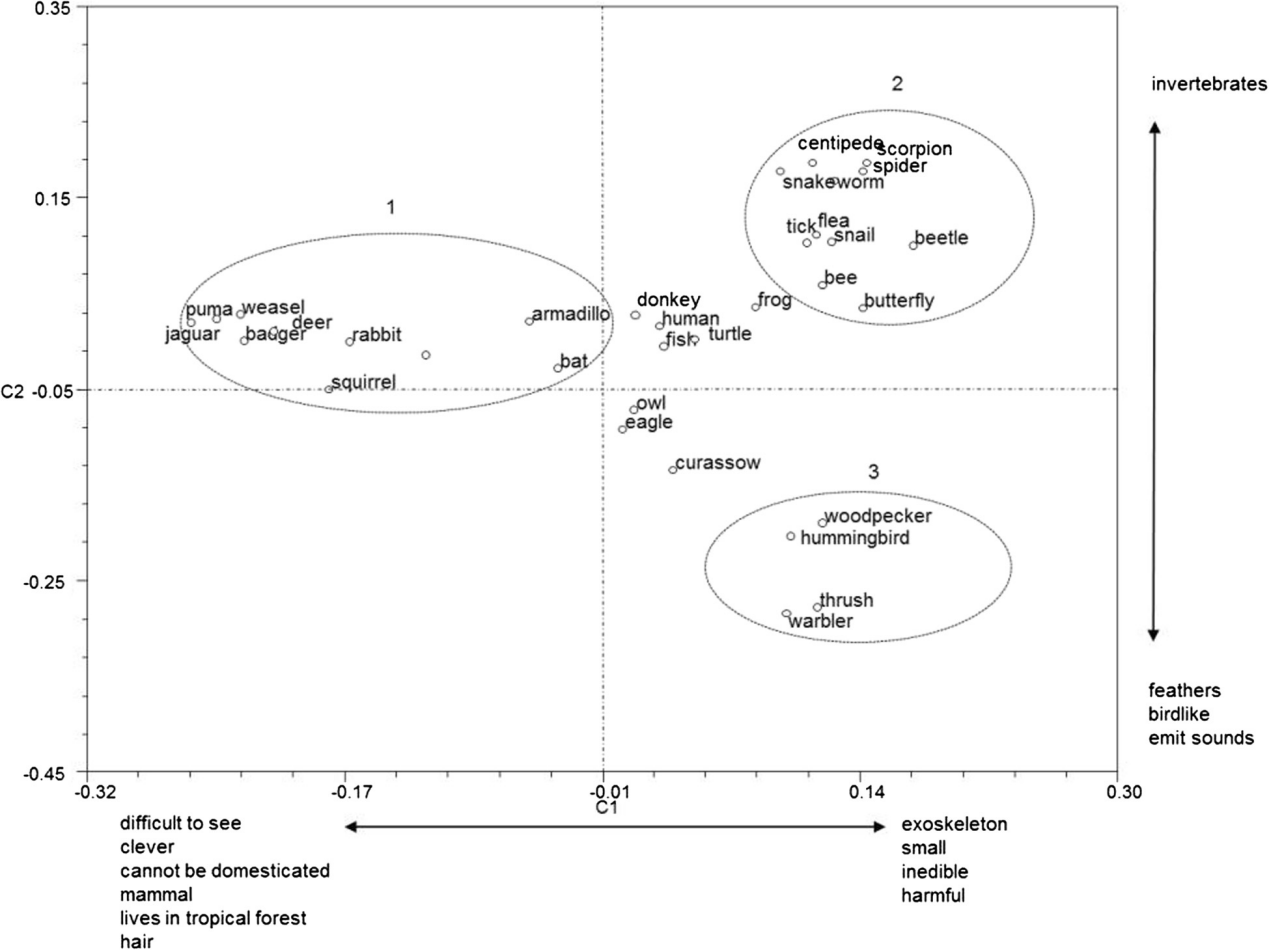
Principal component analysis for all judgments in Cuicatec pile-sorting analysis.

**Figure 5:**
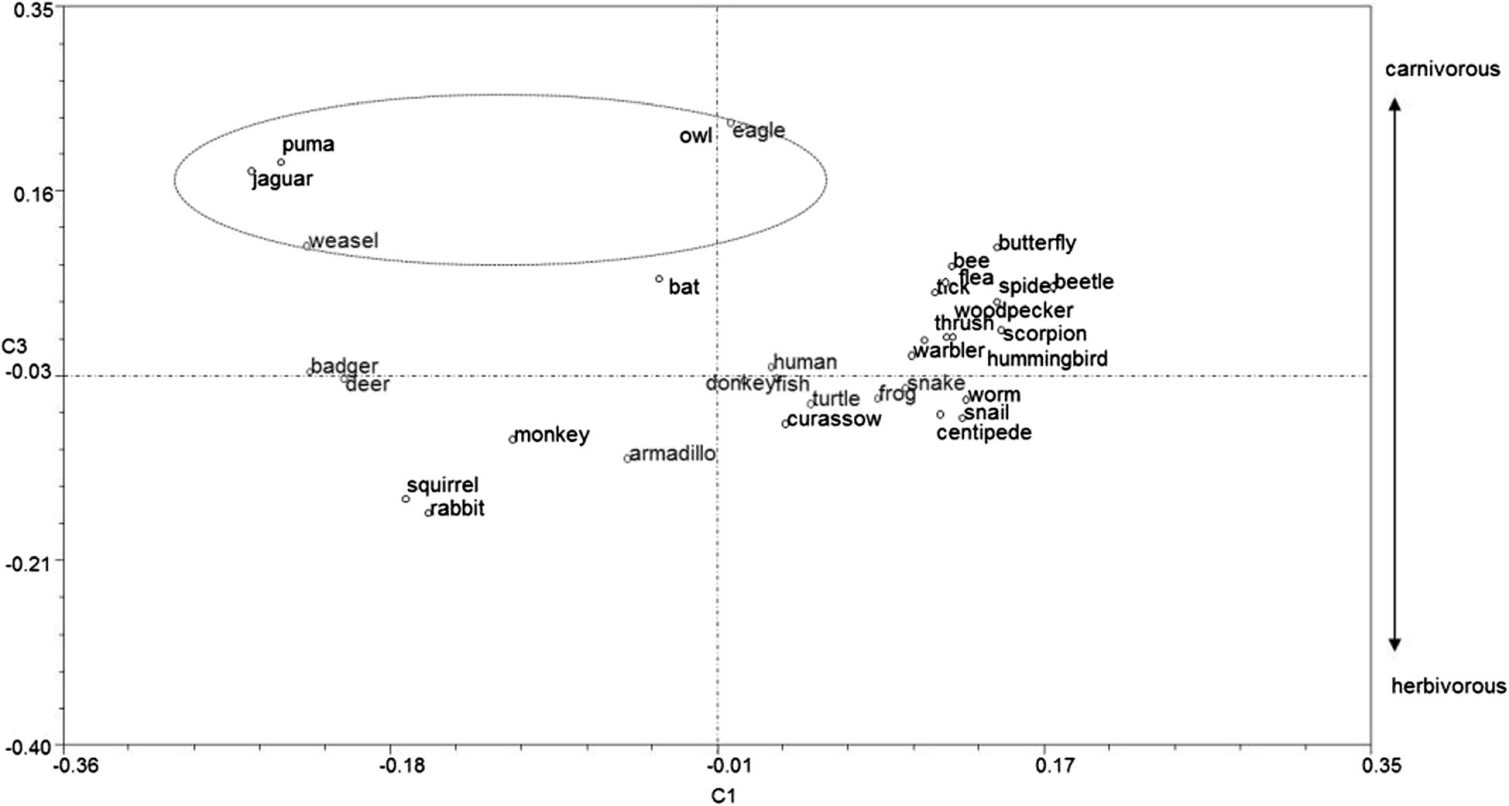
Three-dimensional graph of principal component 3 in Cuicatec pile-sorting analysis.

If we now turn to Cuicatec judgments for all animals, and examine Figure 4, and in particular PC 1 on the X-axis, the main grouping obtained is group 1. This is separated from groups 2 and 3 due to the following high values (Additional file 2): -8872 for character B8 (animals designated as ‘clever’, those that are difficult to see, and those that cannot be domesticated); -0.8234 for character H4 (those animals living in tropical forest), -0.7432 for character H5 (those living in semi-deciduous forest, -0.7680 for character M8 (animals with hair), and −0.7503 for character SC4 (mammals). On the Y axis, PC2 generates group 3 separated from groups 1 and 2 due to the higher values for character B13, animals producing sounds (−0.8109), character M10, animals with feathers (−0.8335), and character SC1, *pajaritos* (−0.7600), that is ‘birds’, though it should be noted that there is no word for ‘birds’ as a whole in either traditional Zapotec or Cuicatec. Group 2 has no high values but is separated as these animals have shells or scales (M4), are very small (M1), inedible and/or harmful (S1). Aquatic or semi-aquatic animals (H1) are also separated, as are humans and donkeys due their symbiotic relationship (S5). If we now examine Figure 5, the highest values for PC 3 are - 6.471 for herbivores (A1) and 0.6389 for carnivores (A2). This relationship is indicated by their appearance in the figure within the ellipse bounded by a broken line. In the Cuicatec PCA the first two components accounted for 25.8686 percent of the variation.

Considering the range of overlapping criteria used for classifying animals using pile-sorts by Zapotec in San Miguel Tiltepec and by Cuicatec in San Juan Teponaxtla, it is difficult to represent their overall system of folk classification as a two-dimensional scheme, or as a conventional taxonomic tree diagram. Although groupings based on morphology are robustly evident in accordance with the Berlinian model, diverse non-morphological criteria were also used by research subjects (especially in Figures 2 and 4), while judgments made in the pile-sorting tests are all interrelated in several, often crosscutting, ways, and vary according to context. For this reason it is a better reflection of how Zapotec and Cuicatec actually think about the affinities between different animals in everyday situations to use an n-dimensional model in which each item or animal is simultaneously in more than one classificatory arrangement. For example, if we take the case of the owl: in the context of its association with humans it is a member of a category of omen animals; in terms of its behaviour it is a member of a category of nocturnal animals; in terms of its feeding habits it is considered a carnivore, and finally if it is judged in terms of its morphological attributes it is considered as an animal that ‘can be either small or large’.

**Figure 6:**
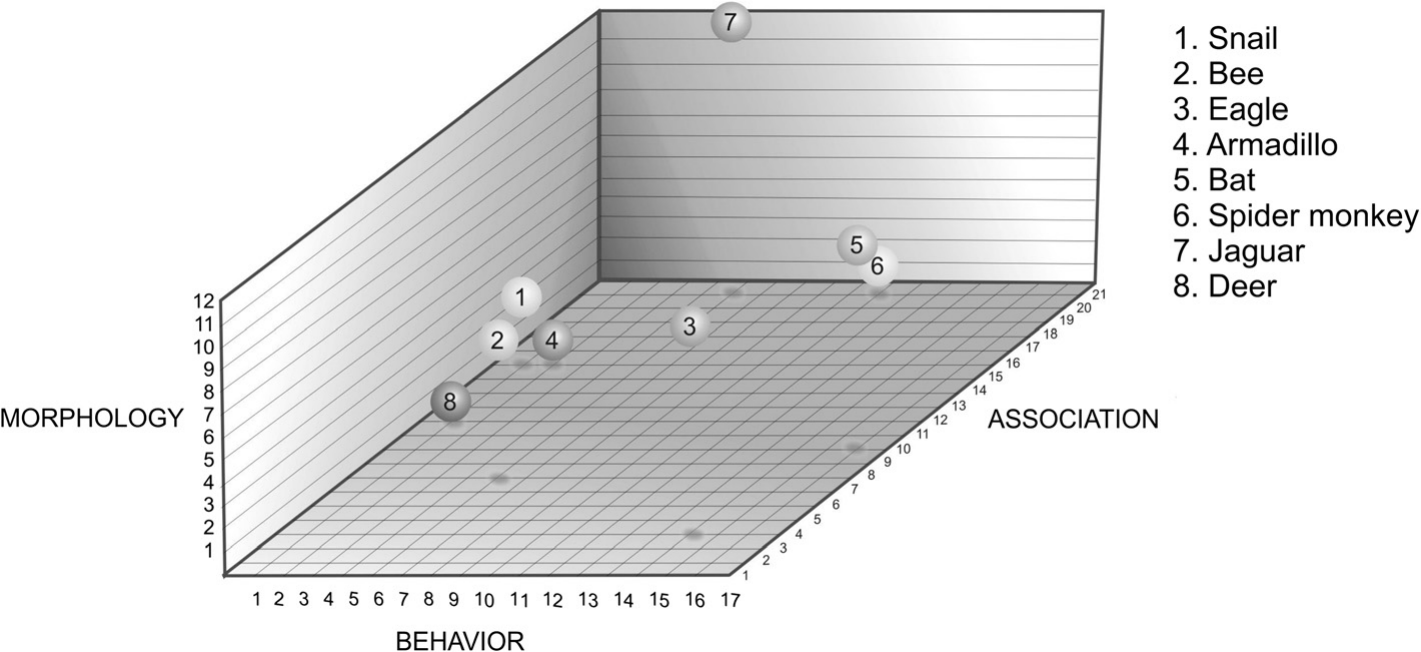
A three-dimensional graph for three Zapotec judgments concerning eight animals, illustrating multidimensional modelling of ethnozoological classification.

For both Zapotec and Cuicatec, zoological classifications are dynamic, varying according to the different contexts in which people refer to or use animals: for example depending on the perceived relationship between humans and animals, in terms of the habitats that they occupy, their alimentary habits or other features of behaviour, or in terms of their morphological characteristics. In order to capture some sense of this dynamic quality, we selected just eight animal types - snail, bee, spider monkey, deer, eagle, armadillo, jaguar and bat - in order to construct an illustrative three-dimensional model. In Figure 6, the X-axis represents judgments concerning ‘behaviour’, the Y-axis ‘morphology’ and the Z-axis ‘association with humans’. We can see from the figure that the values or judgments on each axis are different, for example the eagle has a value of 5 on the Y axis, reflecting its status as an omen animal, a value of 8 on the X axis reflecting its status as a flying animal and a value of 6 on the Z axis, reflecting its status as an animal of great size.

It is impossible to represent all judgments registered in both Zapotec and Cuicatec settlements in a graph of six dimensions for the 33 animals sampled. It would be even more difficult if we were to attempt to represent their ethnozoological classification in this way. In all the groupings produced by both Zapotec and Cuicatec subjects, the great majority of people used just one judgment in order to decide where to place animals in piles, such as ‘animals with four legs’, or ‘animals that are all edible’, ‘animals with hair’. Some people used two judgments, such as: ‘animals that are nocturnal and born from eggs’, ‘animals used in sorcery and that are nocturnal’, and ‘carnivorous animals living in cloud forest’. Some groupings did not appear to be accounted for through one or two simple sorting judgments, and involved more complex reasoning of the kind ‘humans take care of donkeys and rabbits, but the tick is on the donkey’, ‘the jaguar may eat the squirrel, the monkey and the coati, but they share a common habitat in the branches of a tree’, ‘animals with no skeleton, but the spider can eat the bee, butterfly, tick, flea, scorpion and beetle’. Table 5 shows the relationship between age, gender and whether the judgment used in grouping animals in the tests were single, binary or multiple. Multiple judgments were reported for two persons in the Zapotec sample and for four persons in the Cuicatec sample. Overall, subjects tended to sort piles based on single criteria. Age and gender were not shown to influence the results.

**Table 5:** A comparison of the number of judgments used by Zapotec and Cuicatec subjects in grouping animal types in pile-sorting tests, by age and gender

**Zapotec**			**Cuicatec**		
	**Single**			**Single**	
**Children (6–13)**	**Young adults (14–40)**	**Old adults (41–90)**	**Children (6–13)**	**Young adults (14–40)**	**Old adults (41–90)**
2♀-1♂	3♀-3♂	3♀-3♂	4♀-1♂	2♂	3♀-5♂
	**Two**			**Two**	
2♀-1♂	1♀-5♂	2♂	0	1♀-1♂	2♀-5♂
	**Multiple**			**Multiple**	
1♂	1♀	0	0	1♀	2♀-1♂

**Figure 7:**
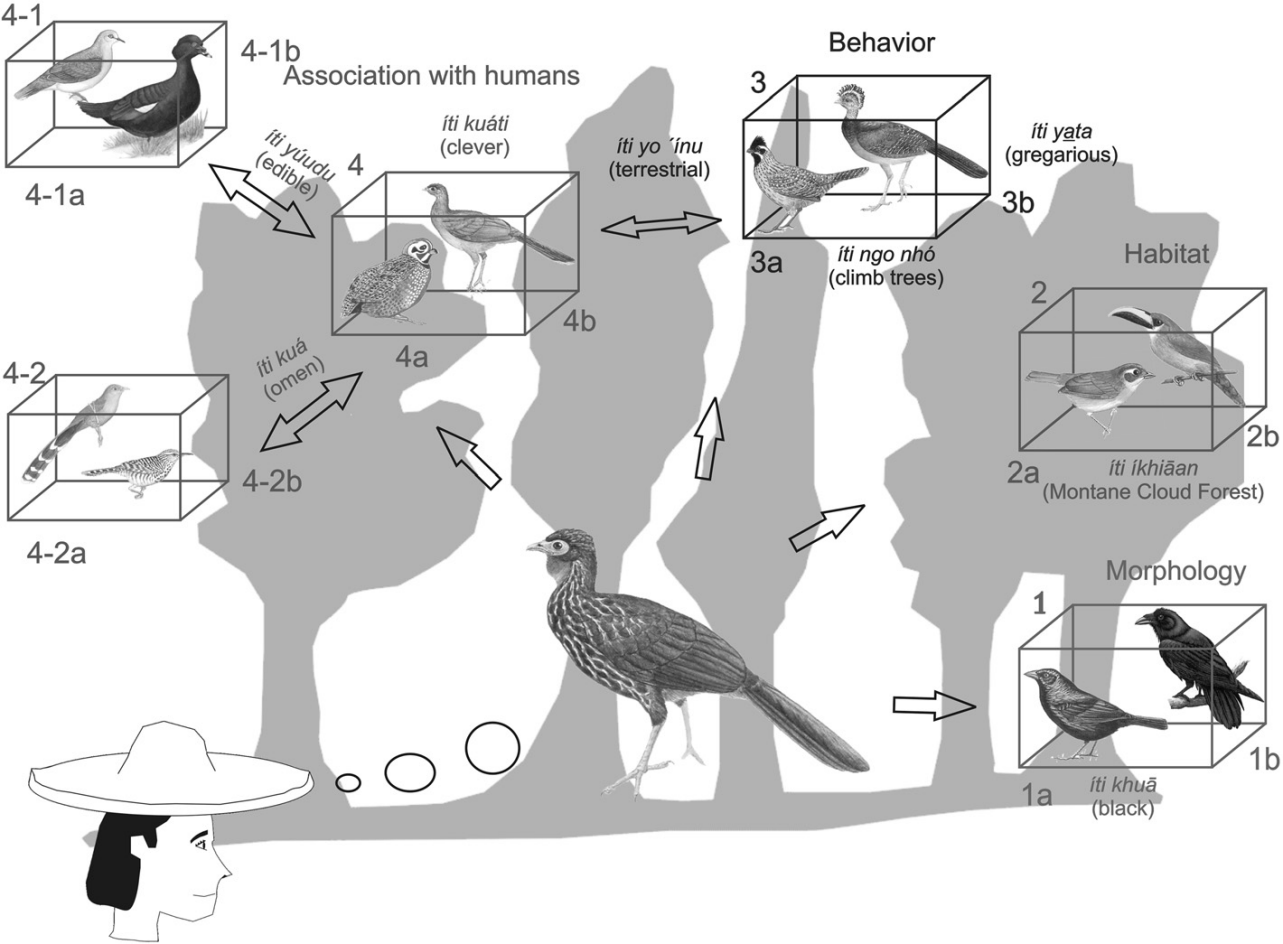
**Classification of *Penelope purpurascens *** **according to different judgments.**

We can also see how multi-dimensionality might be incorporated into the classificatory knowledge of a single species, by referring to Figure 7. In this figure *Penelope purpurascens* (Crested Guan) is classified together with other species depending on different judgments or contexts. These contexts are the basis for the formation of groups, and each group is represented in the figure as a cube, where each side of the cube represents one judgment or context determining location in the same group. In the Zapotec and Cuicatec ethnobiological worlds there exist as many cubes as there are ideas or qualities to locate the connections relating to species. It is useful to hypothesize how an individual person, Zapotec or Cuicatec, thinks about the classificatory affinities of a particular bird species, uninfluenced by the professional concerns of ethnobiologists or conservation biologists. We might imagine that he or she has in mind a series of prototypical images, represented by the contents of each cube in Figure 7. But, as Figure 7 shows, these prototypical images share similarities with other species, depending on the judgments used to form the groups in those cubes. In this example, *Penelope purpurascens* is presented in different ways, depending on the contents of each of the cubes. It is associated with cube 1 on the right hand side of the figure on the basis of colour. *P. purpurascens* is linked with the Crow (*Corvus corax-*1a*)* and the Cowbird (*Molothrus aeneus-*1b*)* because they both have gloomy feathers, although *P. purpurascens* is also linked with the Emerald Toucan (*Aulacorhynchus prasinus*-2b*)* and the Common Bush-tanager (*Chlorospingus ophthalmicus-*2b) in cube 2 since they can all be found together in the same habitat (Cloud Forest). At the same time, *P. purpurascens* can be placed with the Great Curassow (*Crax rubra*-3b) and the Long-tailed Wood-partridge (*Dendrortyx macroura*-3b) in cube 3 as they all share a similar behaviour, in spending most of the time strutting around on the forest floor, but at the same time gregarious. Finally, *P. purpurascens* is linked to the birds inside cube 4 due to similarities in the way in which these species interact with people. All are regarded as ‘smart’, *P. purpurascens*, the Plain Chachalaca (*Ortalis vetula*-4b) and the Montezuma Quail (*Cyrtonyx montezumae*-4a) being perceived as difficult to catch or see, escaping easily from a human presence. In turn, the theme of human interaction links the species, on the one hand in cube 4–1 with the Muscovy duck (*Cairina moschata-*4-1b) and the Red Billed Pigeon (*Patagioenas flavirostris*-4-1b) because all are edible, and on the other hand in cube 4–2 with the Squirrel Cuckoo (*Piaya cayana*-4-2a) and Boucard’s Wren’ (*Campylorhynchus zonatus*-4-2b) as Cuicatec omen animals.

## Conclusions

Although the hierarchical model of Berlin et al. [20] has proved to be a powerful tool for initially organizing data on folk classification of birds, in their everyday lives, thought and language, Zapotec and Cuicatec individual persons group animals in ways that could be better represented differently. Similar observations have been made by Argueta [49] in his work with P’urepecha people in Michoacán, México. While the regularities reported in cognitivist approaches to folk classification of animals may reveal some background widely-shared template for organizing animal knowledge when looked at from a universal-comparative perspective, the organization of day-to-day classificatory knowledge in traditional societies tends to be embedded and ecological [50]. The multidimensional model explored here helps us to capture some of this everyday reality. Hunn [51] too emphasizes that actual folk classifications exhibit ‘irregularities’ that require us to depart from the Berlinian scheme, and notes that examples of the kind described ‘indicate that formal taxonomic structure does not adequately capture the psychological reality of folk biological classification’. However, while Hunn’s main example of such irregularities for the Southern Zapotec are the way many folk generics are ‘unaffiliated’ to any life-form, and folk-specifics unaffiliated at one or more superordinate ranks (e.g. a folk-specific directly included in a life-form); our main observation has been how intermediate level names and categories based on ecology and behaviour cut across the usual life-form boundaries and violate the integrity of so-called ‘natural’ taxonomy.

Given that the kinds of complex groupings described here, rather than the abstract generalizations of ‘natural’ taxonomy, are those most evidently reflected in everyday life situations, it is this perspective on ethno-zoological classification that is, therefore, also most relevant to the requirements of effective animal conservation [52,53]. For instance, Alcántara-Salinas [54] found that Zapotec in San Miguel Tiltepec categorize birds of prey using the terms *bugaka* or *p’jia*. Ten species reported in the area are grouped under these terms, and the relationship between species is regarded by local people as being indivisible and equal. Thus, there is no separation between hawks, eagles or falcons as independent groups; they are all simply described as *bugaka* or *p’jia*. Therefore, if conservationists wish to preserve one particular bird of prey species in this category they will have to consider the relations Zapotec perceive between all species in the category. This kind of information is crucial if the relevant agencies are to implement a more realistic bird conservation strategy in Northern Oaxaca. Moreover, as Bonta [55] recommends on the basis of his experience in Honduras, conservation must not only be based on local traditional knowledge of birds and their management, but traditional knowledge owners must become the co-designers and co-managers of protected areas. This kind of locally embedded conservation strategy must in turn involve recommendations that reflect how indigenous people group species in everyday encounters and practices.

If we are to understand the practical implications of ethnobiological classification, for example as these might influence conservation strategies, we must adopt a perspective that emphasises the complex and often fuzzy categories that people actually use rather than some abstract ‘natural’ and unified scheme that might be inferred from some kinds of analysis. The approaches we have described in this paper show the links between birds and the wider domain of animals, and suggest that it is sometimes misleading to separate out groups of animals defined in terms of macro phylogenetic taxonomic categories (e.g. by Order, Class, Phylum) when all ethnobiological knowledge is connected, even in classifications. By using a multidimensional model we can see how each category can have a different value or position depending on the context in which people refer to it. This model reflects the holistic vision of nature as Zapotec and Cuicatec experience it, and for this reason can be used as a tool in developing conservation strategies with more confidence. Practical interests and functional criteria are intrinsic to the structure of folk classifications used in everyday contexts, and we agree with Morris [16] that ‘folk classifications are inherently complexive rather than hierarchic, and dominated by concrete associations and “functional entailment”’.

## Abbreviations

BCE: Before common era; BP: Before present; CONACYT: National Council of Science and Technology; INALI: The National Institute of Indigenous Languages; INEGI: The National Institute of Statistics and Geography; NTSYS-pc: Numerical taxonomy system-pc software; PCA: Principal component analysis; SERBO: Society for the study of biotic resources of Oaxaca.

## Competing interests

The authors declare that they have no competing interests.

## Authors’ contributions

The fieldwork on which this study is based was undertaken by A-S, who with V-C, also initiated the research project. V-C helped with the Z language and qualitative data analysis. A-S coordinated the work and wrote the first draft of the paper. E has contributed to the discussion of theoretical aspects of folk classifications and has advised on presentation. Caballero supervised A-S in Mexico and helped with, along with V-C, the analysis of quantitative data using the NTSYS program. A has contributed to the review of background literature. All authors have read and approved the final manuscript.

## Supplementary Material

Additional file 1 **Zapotec pile sorting loadings obtained for each main component in the analysis.** Loadings with highest vectors making the groupings along the three principal components are marked*. Click here for file

Additional file 2 **Cuicatec pile sorting loadings obtained for each main component in the analysis.** Loadings with highest vectors making the groupings along the three principal components are marked*. Click here for file
