# Supplementary material for: Alternative ways of representing Zapotec and Cuicatec folk classification of birds: a multidimensional model and its implications for culturally-informed conservation in Oaxaca, México
Source: J Ethnobiol Ethnomed. 2013 Dec 9;9:81. doi: 10.1186/1746-4269-9-81 (PMC4120933; doi:10.1186/1746-4269-9-81)
Supplement: Additional file 1 — Zapotec pile sorting loadings obtained for each main component in the analysis. Loadings with highest vectors making the groupings along the three principal components are marked*. [file 1746-4269-9-81-S1.doc]

Additional file 1. Zapotec pile sorting loadings obtained for each main component in the analysis. Loadings with highest vectors making the groupings along the three principal components are marked*.

| Code | Judgments | C1 | C2 | C3 |
| --- | --- | --- | --- | --- |
| 1) Association with humans | | ------------------------------------------------ | | |
| S1 | negative relationship | 0.5971 | 0.4596 | 0.1744 |
| S2 | positive relationship | −0.7241* | −0.3502 | −0.2699 |
| S3 | negative and positive relationship | −0.1161 | 0.0247 | −0.0530 |
| S4 | omen animal | 0.1493 | −0.6068 | 0.5189 |
| S5 | neutral relationship | 0.2343 | 0.2628 | −0.1000 |
| S6 | unappreciated by humans | −0.1173 | 0.2770 | 0.4314 |
| S7 | appreciated by humans | 0.2868 | 0.3092 | −0.0325 |
| 2) Behavior | | | | |
| C1 | climbs trees | −0.5176 | 0.0220 | 0.1686 |
| C2 | crawls over the ground | 0.4017 | 0.4958 | −0.1183 |
| C3 | Flys | 0.2858 | −0.8243* | 0.0750 |
| C4 | walks and runs | −0.2658 | −0.1565 | −0.3545 |
| C5 | Jumps | −0.0898 | 0.0050 | −0.2361 |
| C6 | difficult to see (clever) | 0.0208 | 0.2386 | 0.1010 |
| C7 | makes holes in trees | −0.1527 | 0.1148 | −0.1249 |
| C9 | digs in the ground | 0.2612 | 0.0343 | −0.0906 |
| U | Solitary | 0.3174 | 0.3549 | −0.0014 |
| G | gregarious and friendly | 0.3251 | −0.2811 | −0.2956 |
| N | Nocturnal | −0.0116 | 0.0202 | 0.8099* |
| D | Diurnal | −0.4454 | 0.0096 | 0.2598 |
| Z | emits sounds and is nocturnal | 0.1524 | −0.0648 | 0.6562* |
| C12 | works hard, smokes, hunts | −0.2971 | 0.0979 | −0.1617 |
| C13 | symbiotic relationship | −0.2839 | 0.0475 | −0.2018 |
| 3) Feeding habits | | | | |
| A1 | herbivorous (grasses and fruits) | −0.2798 | −0.1810 | −0.2951 |
| A2 | Carnivorous | −0.1474 | −0.1807 | 0.6001 |
| A3 | Granivorous | −0.4379 | −0.1740 | −0.2483 |
| A5 | blood sucking | 0.3008 | 0.0454 | 0.3984 |
| A6 | Nectarivorous | 0.1787 | −0.3225 | −0.1870 |
| A7 | part of trophic chain | 0.1458 | 0.0784 | −0.1662 |
| A8 | bug eating | 0.3554 | −0.2095 | −0.1426 |
| A9 | ‘tortilla’ eating | −0.1826 | 0.1476 | −0.1561 |
| 4) Habitat | | | | |
| L1 | aquatic or semiaquatic | −0.0047 | 0.2338 | −0.0952 |
| L2 | cold forest | −0.7247* | 0.3020 | 0.1484 |
| L3 | distant from settlement | −0.1180 | −0.0259 | −0.2623 |
| L4 | in settlements | 0.4048 | 0.2324 | 0.0621 |
| L5 | warm or hot forest | 0.3614 | 0.3806 | −0.0862 |
| L7 | lives all around | 0.1486 | −0.2895 | 0.0109 |
| L8 | inside flowers | 0.2704 | −0.1217 | −0.1075 |
| L9 | on dry land | −0.4851 | 0.1843 | −0.0363 |
| L10 | in caves | −0.1604 | 0.0493 | 0.4475 |
| 5) Morphological attributes | | | | |
| M1 | Small | 0.6291 | 0.2757 | −0.1674 |
| M2 | Hairy | −0.4224 | 0.6104 | 0.1869 |
| M3 | similar ears | −0.3186 | −0.0755 | −0.2487 |
| M4 | similar hands | 0.5594 | 0.1835 | −0.1342 |
| M5 | similar form | 0.0289 | −0.7870* | −0.2153 |
| M6 | similar colour | 0.5096 | 0.4176 | −0.1517 |
| M8 | big ears | 0.0910 | −0.2122 | 0.6862 |
| M9 | similar kinds of leg | 0.3383 | 0.3233 | −0.1283 |
| M10 | four legs | −0.7931* | 0.2766 | 0.0704 |
| M11 | with feathers | 0.0963 | −0.4124 | −0.0053 |
| 6) Other | | | | |
| P2 | ‘they just are’ | −0.7903* | 0.4114 | −0.0522 |
| P10 | Bird | 0.0802 | −0.8442* | 0.0647 |
