# Supplementary material for: Alternative ways of representing Zapotec and Cuicatec folk classification of birds: a multidimensional model and its implications for culturally-informed conservation in Oaxaca, México
Source: J Ethnobiol Ethnomed. 2013 Dec 9;9:81. doi: 10.1186/1746-4269-9-81 (PMC4120933; doi:10.1186/1746-4269-9-81)
Supplement: Additional file 2 — Cuicatec pile sorting loadings obtained for each main component in the analysis. Loadings with highest vectors making the groupings along the three principal components are marked*. [file 1746-4269-9-81-S2.doc]

Additional file 2. Cuicatec pile sorting loadings obtained for each main component in the analysis. Loadings with highest vectors making the groupings along the three principal components are marked*.

| Code | Judgments | C1 | C2 | C3 |
| --- | --- | --- | --- | --- |
| 1) Association with humans | | ------------------------------------------------- | | |
| S1 | negative relationship | 0.1930 | 0.5089 | 0.2044 |
| S2 | positive relationship | −0.0634 | −0.5728 | −0.4398 |
| S3 | negative and positive relationship | −0.0249 | −0.4418 | −0.2681 |
| S4 | neutral relationship | 0.4796 | 0.0351 | 0.0180 |
| S5 | symbiotic with humans | 0.0300 | 0.0510 | −0.0730 |
| S6 | omen animal | −0.2738 | −0.0679 | 0.2439 |
| S7 | humans placed in solitary group | 0.0338 | 0.0264 | −0.0348 |
| S8 | genetically related to humans | −0.1658 | −0.0110 | −0.2527 |
| S9 | companion animal | −0.2249 | −0.0321 | −0.2811 |
| 2) Behavior | | | | |
| B1 | climbs trees | −0.1471 | 0.2454 | −0.3271 |
| B2 | crawls over the ground | 0.1689 | 0.2940 | −0.3726 |
| B3 | digs in the ground | 0.1535 | 0.4269 | −0.3527 |
| B4 | Flys | 0.4183 | −0.6769 | 0.3395 |
| B5 | sticks on clothing | 0.2427 | 0.2020 | 0.1682 |
| B6 | walks and runs | 0.0153 | −0.0398 | −0.1847 |
| B7 | Jumps | −0.0348 | 0.0474 | −0.2863 |
| B8 | difficult to see, clever, can be domesticated | −0.8872* | 0.0146 | 0.3914 |
| B9 | cannot be domesticated | −0.2249 | −0.0321 | −0.2811 |
| B10 | makes holes in trees | 0.1659 | −0.3045 | 0.0315 |
| B11 | Gregarious | 0.2686 | −0.7496 | 0.0252 |
| B12 | Nocturnal | −0.0149 | −0.1044 | 0.2340 |
| B13 | emits sounds | 0.2983 | −0.8109* | 0.0307 |
| B14 | sleeps in hanging position | −0.1963 | −0.0540 | −0.1128 |
| B15 | Oviparous | −0.0265 | −0.1068 | 0.2240 |
| B16 | Migrates | 0.2217 | −0.6756 | 0.0150 |
| 3) Feeding habits | | | | |
| A1 | Herbivorous | −0.5752 | −0.0082 | −0.6471* |
| A2 | Carnivorous | −0.5732 | −0.0113 | 0.6389* |
| A3 | bug eating | 0.3979 | −0.3730 | −0.1145 |
| A4 | blood sucking | 0.1451 | 0.1839 | 0.1738 |
| A5 | Nectarivorous | 0.1820 | −0.1035 | 0.1348 |
| A6 | eats food waste and earth | 0.4059 | 0.5878 | −0.1229 |
| A7 | Omnivorous | −0.6313 | 0.0744 | 0.5225* |
| A8 | part of trophic chain | 0.0972 | 0.4292 | 0.4510 |
| A9 | Granivorous | −0.3488 | −0.0350 | −0.5470* |
| 4) Habitat | | | | |
| H1 | aquatic or semiaquatic | 0.1471 | 0.0802 | −0.1700 |
| H2 | Terrestrial | 0.2434 | 0.5694 | −0.2864 |
| H3 | cloud forest | −0.5138 | 0.0178 | 0.0448 |
| H4 | tropical forest | −0.8234* | −0.1382 | −0.2063 |
| H5 | semi-deciduous forest | −0.7432* | 0.0218 | −0.2519 |
| H6 | distant from settlement | 0.4455 | 0.3398 | 0.1237 |
| H7 | can be bought in the city | −0.3132 | 0.0178 | −0.1021 |
| H8 | under stones | 0.3231 | 0.5386 | −0.0829 |
| 5) Morphological attributes | | | | |
| M1 | Small | 0.4203 | 0.3907 | 0.3213 |
| M2 | similar form | −0.3882 | 0.0311 | 0.0182 |
| M3 | Vermiform | 0.2412 | 0.4201 | −0.1742 |
| M4 | with shell or scales | 0.0497 | 0.0380 | −0.2684 |
| M5 | Invertebrate | 0.4755 | 0.4643 | 0.2863 |
| M6 | four legs | −0.5551 | 0.0590 | 0.5838* |
| M8 | Hairy | −0.7680* | −0.0036 | −0.0750 |
| M9 | Slimy | 0.2186 | 0.2247 | −0.1802 |
| M10 | with feathers | 0.2790 | −0.8335* | 0.0585 |
| 6) Other | | | | |
| SC1 | Bird | 0.3077 | −0.8573* | 0.0652 |
| SC2 | Rodent | −0.3499 | −0.0199 | −0.5945* |
| SC3 | Eagle | 0.0024 | −0.1318 | 0.1890 |
| SC4 | Mammal | −0.7503* | 0.0625 | 0.2325 |
| SC5 | ‘rabbit is grandchild of the deer’ | −0.3888 | 0.0127 | −0.3628 |
| SC6 | not grouped in any judgment | 0.0334 | 0.0752 | 0.1251 |
